# Chicken Protein S Gene Regulates Adipogenesis and Affects Abdominal Fat Deposition

**DOI:** 10.3390/ani12162046

**Published:** 2022-08-11

**Authors:** Lijin Guo, Weiling Huang, Siyu Zhang, Yulin Huang, Yibin Xu, Ruiquan Wu, Xiang Fang, Haiping Xu, Qinghua Nie

**Affiliations:** 1 Lingnan Guangdong Laboratory of Modern Agriculture & State Key Laboratory for Conservation and Utilization of Subtropical Agro-Bioresources, Department of Animal Genetic Breeding and Reproduction, College of Animal Science, South China Agricultural University, Guangzhou 510642, China; 2 Guangdong Provincial Key Laboratory of Agro-Animal Genomics and Molecular Breeding, and Key Laboratory of Chicken Genetics, Breeding and Reproduction, Ministry of Agriculture, Guangzhou 510642, China

**Keywords:** *PROS1*, chicken, adipogenesis, abdominal fat deposition, association

## Abstract

**Simple Summary:**

Low-fat meat is increasingly desired by the public due to the growing popularity of healthy diets, and the excessive accumulation of abdominal fat increases costs in the broiler breeding industry, all of which have encouraged breeding changes in the broiler industry. Investigating fat accumulation at a cellular level from a genetic perspective will help us understand gene-mediated abdominal fat accumulation in chickens. This study aimed to explore the role of the *PROS1* gene in adipose cells and its application prospect in broiler breeding. Based on our findings, we found that the *PROS1* gene can contribute to adipose cell proliferation and can reduce fat deposits at the cellular level, and its mutations are highly correlated with chicken fat traits.

**Abstract:**

(1) Background: Excessive abdominal fat deposition in broilers not only causes feed waste but also leads to a series of metabolic diseases. It has gradually become a new breeding goal of the broiler industry to improve growth rates and to reduce abdominal fat rates. In a previous study, *PROS1* was highly expressed in low-abdominal fat broilers, suggesting a potential role in broilers adipogenesis. However, the function of *PROS1* in preadipocytes and its association with abdominal fat traits need to be characterized. (2) Methods: qRT-PCR and Western Blot were used to quantify gene expression at the RNA and protein levels; flow cytometry and EdU were carried out to detect cell proliferation; and a GLM analysis was used to determine the association between *PROS1* SNPs and carcass traits. (3) Results: *PROS1* was downregulated in high-abdominal fat chicken; *PROS1* contributed preadipocyte proliferation but suppressed preadipocyte differentiation; and the SNPs in the *PROS1* 5′ flank were significantly associated with the abdominal fat weight rate. (4) Conclusions: Chicken *PROS1* is able to suppress adipogenesis, and its polymorphisms are associated with the abdominal fat weight rate, which can be considered the molecular markers for chicken breeding, indicating that *PROS1* is an effective potential gene in regulating abdominal fat deposition.

## 1. Introduction

As an important agricultural animal, chicken provides most of the world’s population with major sources of protein such as meat and eggs [1]. In addition to this, chicken is an important model organism that bridges the evolutionary gap between mammals and other vertebrates [2,3]. Modern commercial broilers are products that have been intensively selected for their rapid growth and improved muscle mass for generations [4]. This selection, based on a fast-growing phenotype, has also led to the unsatisfactory problem of excessive abdominal fat deposition in chickens [5,6]. The characteristics of abdominal adipose tissue are also closely associated with the meat quality of chickens [7]. There are many studies on the growth and development of chicken skeletal muscle, but less is known about the genetic mechanisms by which fat-related genes regulate chicken fat deposition. It was reported that transcriptional factor peroxisome proliferator-activated receptor gamma (PPARγ) is a very important regulator in chicken preadipocyte differentiation [8]. Additionally, multiple regulators have been identified to control chicken adipogenesis by regulating the PPARγ signal pathway [9,10]. *FNIP2* was able to upregulate the PPARγ signal pathway to promote preadipocyte differentiation and to further intensify lipid biosynthesis [9]. Moreover, lncRNA also plays an important role in the proliferation of adipocytes [11]. Although a few studies have contributed to chicken preadipocyte proliferation and differentiation, further studies on their molecular mechanisms would provide more complete insights into chicken abdominal adipose deposition.

PROS1, also known as Protein S, is a well-known gene that encodes a vitamin K-dependent plasma protein. This protein acts as a cofactor of anticoagulant protease, activated protein C (APC), to inhibit blood coagulation [12]. It is present in plasma in either a free, functionally active form or in an inactive form bound to the C4b-binding protein [13]. Over the last few decades, studies concerning PROS1 have focused mainly on its involvement in human cardiovascular disease, with few reports mentioning the role of PROS1 in obesity. A study about mechanisms of weight maintenance under high- and low-protein, low-glycemic index diets revealed that PROS1 levels were indicative of successful body weight maintenance [14]. However, no molecular mechanism of PROS1 regulating adipogenesis has been elucidated so far, whether human, mouse or chicken models. In our preliminary work, we conducted a transcriptome sequencing (PRJNA656618) of abdominal fat in 100-day-old Sanhuang broilers and found that *PROS1* mRNA was highly expressed in low-fat individuals, suggesting that *PROS1* may have an antagonistic effect on abdominal fat deposition in chickens.

## 2. Materials and Methods

### 2.1. Animal Experiments and Ethics Statement

An F2 resource population (Mahuang chickens, *n* = 288) with growth traits was used in an association analysis between *PROS1* SNPs and growth traits. Additionally, all animal experiments in this research were approved by the Animal Welfare Committee of South China Agricultural University (Approved ID: SCAU#2021F074). All animal experiments were conducted complying with the regulations of the People’s Republic of China on the control of experimental animals.

### 2.2. Cell Culture

DMEM/F12 medium (Gibco, CA, USA) with 15% fetal serum (Gibco, CA, USA) and 1% streptomycin/penicillin (Invitrogen, CA, USA) was used to cultured immortalized chicken precursor adipocytes (ICP-1) at 37 °C with 5% CO_2_. When the cells grew to 70% confluence, they were transfected with plasmids or oligonucleotides. Lipofectamine 3000 kit (Invitrogen, CA, USA) was used for transfection according to the manufacturer’s instructions. For preadipocyte differentiation induction, the growth medium with 0.2% oleic acid (Sigma, CA, USA) was used when the cells grew to 90% confluence.

5-ethynyl-2′-deoxyuridine (EdU) assay was used to detect cell proliferation activity by using a Cell-Light EdU Apollo567 In Vitro Kit (RiboBio, Guangzhou, China). After 48 h of transfection, the cell culture medium was added to 20 μM 5-ethynyl-20-deoxyuridine (RiboBio, Guangzhou, China) for 2 h of incubation in a cell incubator. Subsequently, the cells were stained with an Apollo567 staining agent and with a Hoechst staining agent after they were washed. Finally, the random views of the cells were captured using a DMi8 microscope (Leica, Wetzlar, Germany). The rate of EdU-stained cells was counted using ImageJ software (1.53k version).

For cell glucose content measurement, the cells were fixed using ORO Fixative (Solarbio, Beijing, China) for 30 min at room temperature after washing. Then, the cells were embathed using 60% isopropanol (Damao, Tianjin, China). ORO Stain agent (Solarbio, Beijing, China) was used for lipid droplet staining for 20 min at room temperature. Finally, the redundant staining agent was washed by water for five times. The isopropanol was used for oil red elution and to transfer the oil red onto a 96-well plate. The absorbance at 450 nm was measured in a Microplate Reader (BioTek, Winooski, VT, USA).

For glycerol content measurement, the cells were collected by 0.25%-EDTA trypsin (Gibco, Carlsbad, CA, USA). The cells were crushed by ultrasonic waves, and then, they were incubated in 95 °C for 20 min. After incubation, they were centrifuged in 8000× *g*, 25 °C for 10 min. The supernatant was transferred into a new tube for subsequent glucose content and triglyceride content detection. For glucose content measurement, the Glucose Content Kit (Solarbio, Beijing, China) was used following its manufacturer’s protocol. Finally, the absorbance at 620 nm was measured in a Microplate Reader (BioTek, Winooski, VT, USA).

For glycerol content measurement, the Triglyceride Content Detection Kit (Jiancheng, Nanjing, China) was used by following its manufacturer’s protocol. The absorbance at 510 nm was measured in a Microplate Reader (BioTek, Winooski, VT, USA).

For flow cytometry analysis of cell cycle, ICP-1 was collected by using 0.25%-EDTA trypsin (Gibco, Carlsbad, CA, USA) after a 48 h transfection and then fixed in 70% ethanol (Damao, Tianjin, China) for 16 h at −20 °C. Next, centrifugation was performed at 2000× *g*, 4 °C for 5 min to collect cells from the cell suspension, and 1 mL of ice-cold PBS (Gibco, Carlsbad, CA, USA) was used to wash the cell sediment and cells were stained with 0.5 mL of Propidium iodide (PI) solution (Beyotime, Shanghai, China) at 37 °C in the dark for 30 min. Finally, the cells were analyzed by a CytoFlex instrument (Beckman, Brea, CA, USA).

### 2.3. Reverse Transcription Reaction and Quantified Real-Time PCR (qRT-PCR)

The MagZol Reagent (Magen, Guangdong, China) was used to extract total RNA following the manufacturer’s protocol. The total RNA was used to synthesize cDNA by HiScript II Q RT SuperMix for qPCR (+gDNA wiper) (Vazyme, Nanjing, China) according to the manufacturer’s protocol. Quantitative PCR (qPCR) was performed using ChamQ Universal SYBR qPCR Master Mix (Vazyme, Nanjing, China) on ABI QuantStudio 5 instrument (Thermo Fisher, Grand Island, NY, USA). The primers used in qRT-PCR were designed by Primer Premier 6.0 software and synthesized by TSINGKE (Beijing, China). The primers are listed in Table S1.

### 2.4. Plasmid Construction and siRNA Synthesis

The complete CDS of *PROS1* (XM_040658752) was cloned onto the XhoI and XbaI sites in the pcDNA3.1 vector (Promega, Madison, WI, USA). Plasmid extraction was conducted using a HiPure Plasmid EF Mini Kit (Magen, Guangzhou, China) following its manufacturer’s protocol. The specific siRNA (5′-GTAAGAATACCCTGGGAAA-3′) of *PROS1* was designed and synthesized by RiboBio (Guangzhou, China).

### 2.5. Western Blot

The protein was extracted from the cells using an ice-cold radio immunoprecipitation (RIPA) lysis buffer (Beyotime, Shanghai, China) with 1 mM phenylmethyl sulfonyl fluoride (Biosharp, Hefei, China). The protein was separated in a 10% SMART-PAGETM Precast Protein gel (SMART, Changzhou, China) under 120 V for 60 min. The protein was transferred onto a polyvinylidene fluoride (PVDF) membrane (BioRad, Hercules, CA, USA). The quick block liquid was used for blocking for 20 min at room temperature. Then, the membrane was incubated with anti-PPARγ (1:1000; bs-0530R, BIOSS), anti-CEBPα (1:1000; LS-B4685, LABIO), or anti-GAPDH antibodies (1:2000; bsm-33033M, BIOSS) at 4 °C overnight. Subsequently, anti-mouse IgG HRP-secondary antibody (1:10,000; 7076P2, CST) or anti-rabbit IgG HRP-secondary antibody (1:10,000; 7074P2, CST) were used to labeled the antibody from mouse or rabbit species. Chromogenic reaction was conducted using an ECL Peroxidase Color Development Kit (Vazyme, Nanjing, China) according its manufacturer’s protocol. Subsequently, protein bands were developed in a cut-out sensitive film in an X-ray Photography Box (YishengBio, Shanghai, China). The protein brands in film were captured in an Odyssey instrument (Li-cor, Lincoln, NE, USA). The gray level was quantified in ImageJ software.

### 2.6. Polymorphisms in PROS1 and Their Associations with Growth Traits

The 288 DNA samples were used for PCR, and the PCR products were sent for Sanger sequencing in TSINGKE (Guangzhou, China). SnapGene software (6.0.2 version) was used to identify the SNPs of all individuals. The genotypes were divided according to SNPs; then, the allele frequency was counted; and whether they conformed to the Hardy–Weinberg equilibrium was determined. SNPs in accordance with the Hardy–Weinberg equilibrium were analyzed for their associations with growth traits in SPSS 25.0 software based on the following model:

Y = μ + G + D + H + S + e

where Y represents the traits’ phenotypic values, μ is the population mean, G is the effect of genotype, S is the effect of sex, D is the random effect of the dam, and e is the random residual.

### 2.7. Data Statistics and Analysis

The results in this study are presented as means ± SEM, and the difference between the groups was analyzed by student’s t-test, with *: *p* < 0.05. Pearson’s correlation coefficient was used to analyze the correlation among growth traits, and correlation significance was analyzed by a two-tailed test, with the confidence interval at 95% and with *: *p* < 0.05. The LSD test was used to analyze the significance of associations between traits with SNPs, and *p* < 0.05 was considered the criterion. The traits’ difference among genotypes was analyzed by One-Way ANOVA; significant differences are shown in superscript letters, with different letters representing significant differences.

## 3. Results

### 3.1. Aberrant Expression of PROS1 in Broilers with High Abdominal Fat Rate

**Figure 1:**
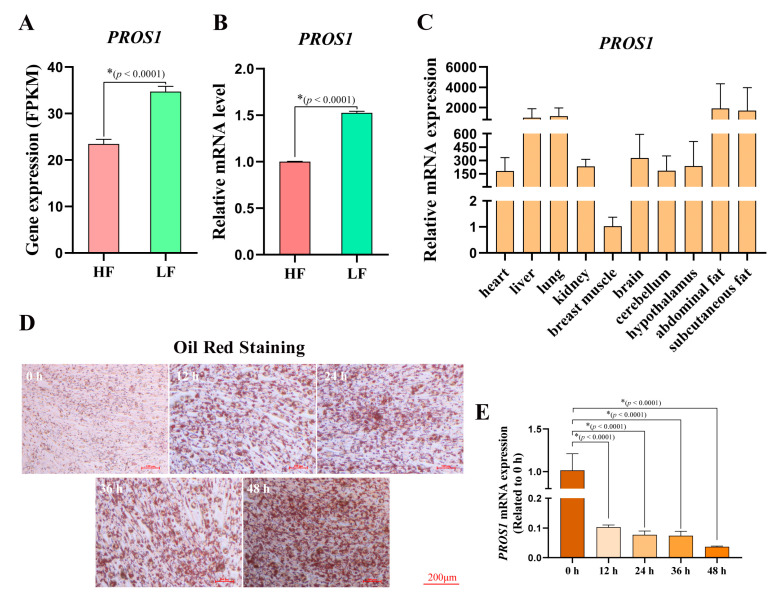
*PROS1* was downregulated in high-fat chicken. (**A**) The gene expression difference of *PROS1* in abdominal fat tissues between high-fat chicken and low-fat chicken shown in our previous RNA-seq. (**B**) The mRNA expression difference of *PROS1* between high-fat chicken and low-fat chicken was detected by qRT-PCR. (**C**) *PROS1* expression profile in different tissues. (**D**) The oil red O staining of ICP-1 cells treated with differentiation induction at different time points. (**E**) The *PROS1* mRNA expression in ICP-1 cells treated with differentiation induction at different time points. The results are all presented as means ± SEM, * *p* < 0.05.

An accumulation of abdominal fat in chickens is correlated with changes in diet and causes great economic loss to the chicken industry. In our previous study [15], transcriptome sequencing was performed for high and low abdominal fat rates in 100-day-old Sanhuang broilers (accession ID: PRJNA656618), and we found that *PROS1* was aberrantly upregulated in the abdominal fat tissues of the low-abdominal fat broilers (Figure 1A). qPCR validation was performed to confirm the difference in mRNA expression of *PROS1* in broilers’ abdominal fat between a high abdominal fat ratio and a low abdominal fat ratio (Figure 1B). We constructed a *PROS1* expression profile, and it was found that *PROS1* was upregulated in fat tissues, including abdominal fat and subcutaneous fat (Figure 1C). The mRNA expression difference suggests that *PROS1* may be involved in the fat deposition process in broilers. In addition, we induced the differentiation of ICP-1 cells from preadipocytes to adipocytes. Here, we measured the lipid droplet formation during preadipocyte differentiation using an oil red analysis, and it can be clearly observed that the number of droplets increased during the differentiation process (Figure 1D). The cells were collected for RNA extraction at four time points (12 h, 24 h, 36 h, and 48 h). It is evident that the expression of *PROS1* declined substantially during the process of ICP-1 cell differentiation (Figure 1E). This evidence drew our attention to the potential role of *PROS1* in the chicken adipogenesis process.

### 3.2. PROS1 Contributes to Chicken Preadipocyte Proliferation

**Figure 2:**
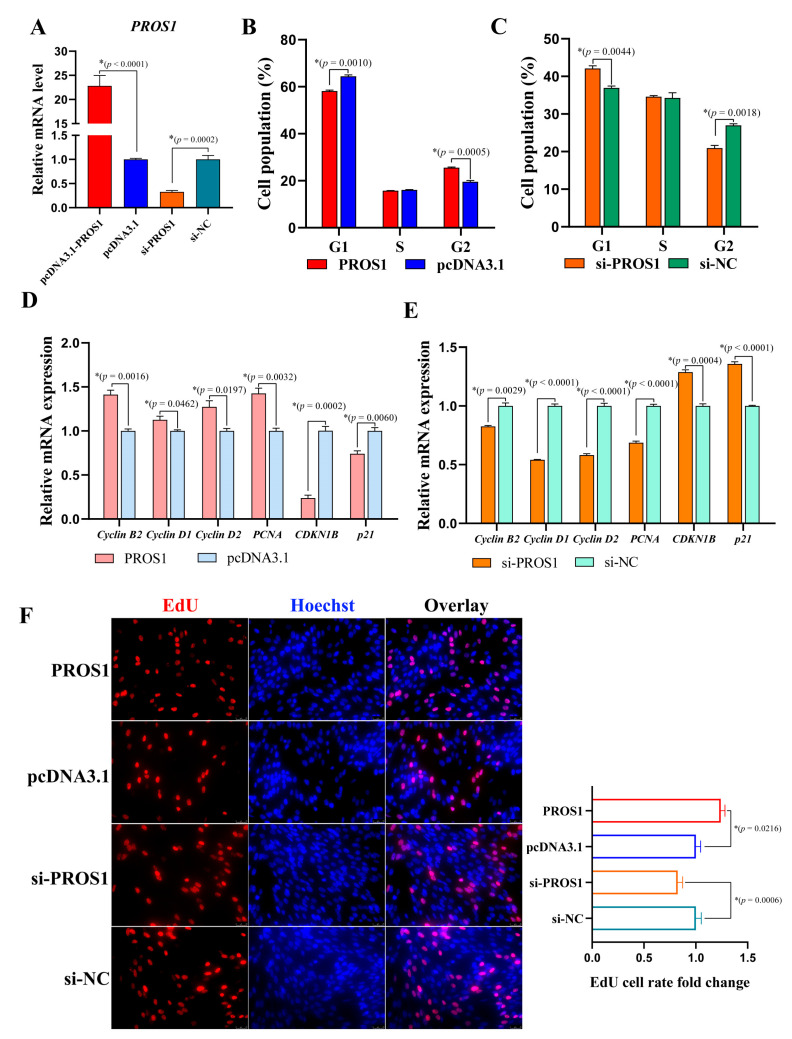
*PROS1* facilitates chicken preadipocyte proliferation. (**A**) The overexpression efficiency or knockdown efficiency of pcDNA3.1-*PROS1* or si-*PROS1*. (**B**,**C**) The flow cytometry analysis of ICP-1 treated with *PROS1* overexpression and knockdown. (**D**,**E**) The mRNA levels of cycle-related genes in ICP-1 treated with *PROS1* overexpression and knockdown were detected by qTR-PCR. (**F**) The EdU staining and statistics of ICP-1 treated with *PROS1* overexpression and knockdown. The results are all presented as means ± SEM, * *p* *<* 0.05.

In order to verify the specific role of *PROS1* in preadipocyte, we constructed an overexpression plasmid and synthesized the siRNA to overexpress or knock down *PROS1*. The transfection efficiency of the pcDNA3.1-*PROS1* plasmid and si-*PROS1* in ICP-1 cells was validated by qPCR. Additionally, it was shown that the *PROS1* mRNA expression could be upregulated more than 50 times and be knocked down by about 70% (Figure 2A). Flow cytometry analysis and EdU assay were conducted to verify whether *PROS1* is able to affect preadipocyte proliferation. It was shown that *PROS1* could accelerate the cell proportion from G1 phase to G2 phase (Figure 2B,C), suggesting a positive effect of *PROS1* in preadipocyte proliferation. We also detected some cell cycle-related gene expression at the mRNA level by qPCR. The mRNA expression of cell cycle-promotion genes (including *Cyclin B2*, *Cyclin D1*, *Cyclin D2* and *PCNA*) and cell cycle-inhibition genes (including *p21* and *CDKN1B*) was severely increased or decreased by *PROS1* (Figure 2D,E). Moreover, the EdU assay results showed that the EdU cell rate was upregulated by *PROS1* (Figure 2F,G), which more intuitively indicates that *PROS1* contributes to preadipocyte proliferation.

### 3.3. PROS1 Suppresses Chicken Preadipocyte Differentiation

**Figure 3:**
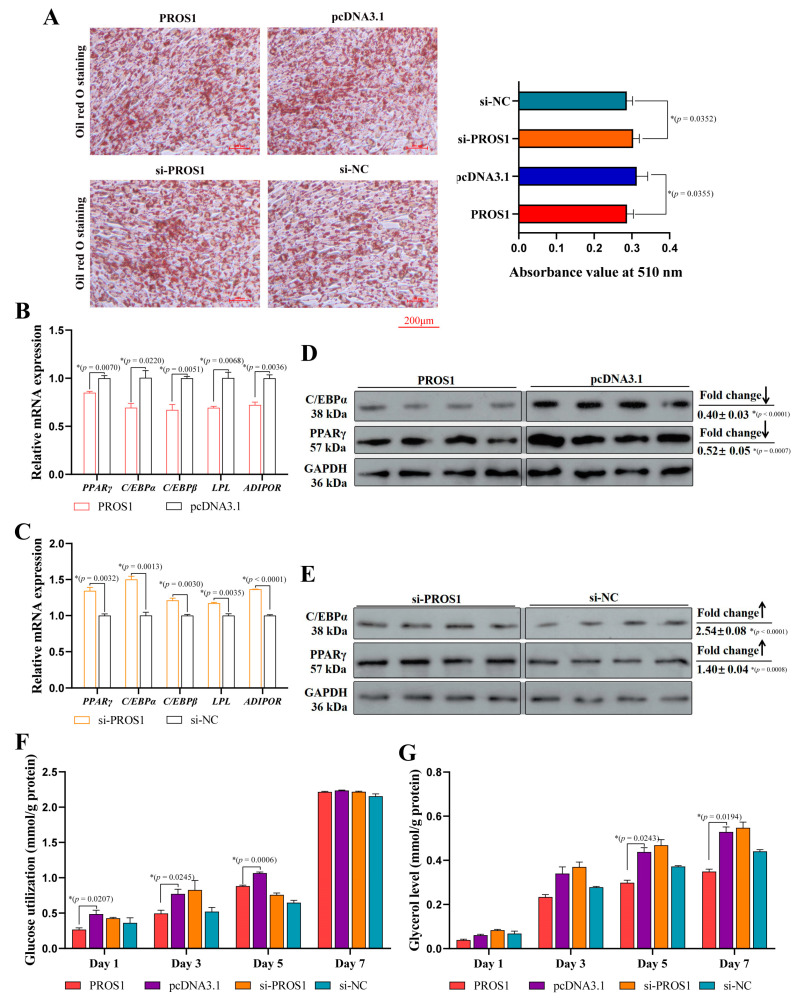
*PROS1* inhibits chicken preadipocyte differentiation. (**A**) The oil red O staining and statistics of ICP-1 after treating with pcDNA.1-*PROS1* plasmid and si-*PROS1*. (**B**,**C**) The mRNA levels of preadipocyte differentiation-related genes in ICP-1 treated with *PROS1* overexpression and knockdown were detected by qRT-PCR. (**D**,**E**) The protein levels of CEBPα and PPARγ in ICP-1 treated with *PROS1* overexpression and knockdown were detected by Western blot. (**F**) The glucose consumption measure during ICP-1 differentiation. (**G**) The glycerol production measure during ICP-1 differentiation. The results are all presented as means ± SEM, * *p* *<* 0.05. Original western blot figures in Figure S1.

Based on the expression difference of *PROS1* in preadipocytes before and after differentiation, we speculated that *PROS1* may be involved in the regulation of the chicken preadipocyte differentiation process. We performed differentiation induction in preadipocytes treated with *PROS1* overexpression or knockdown. After 48 h of the induction treatment, an oil red O assay was performed. As shown in Figure 3A, a high expression of *PROS1* inhibited lipid droplet formation, while the knockdown of *PROS1* promoted lipid droplet formation. The qPCR results showed that the mRNA expression of preadipocyte differentiation-related genes (including PPARγ, CEBPα, CEBPβ, LPL, and ADIPOR) was downregulated by *PROS1*, while the low expression of *PROS1* increased their mRNA expression (Figure 3B,C). In addition, we performed Western blot to verify whether *PROS1* has a regulatory effect on CEBPα and PPARγ protein expression. *PROS1* significantly decreased the CEBPα and PPARγ protein levels, which indicates an inhibiting effect of PPARγ pathway activation (Figure 3D). The low expression of *PROS1* increased the CEBPα and PPARγ protein levels, suggesting that the low expression of *PROS1* contributes to PPARγ pathway activation (Figure 3E). PPARγ pathway activation is involved in glucose metabolism and glycerol metabolism during preadipocyte differentiation. Therefore, we measured the amount of glucose utilization and glycerol production. Glucose consumption increases with the preadipocyte differentiation time. The preadipocytes treated with pcDNA3.1-*PROS1* plasmid showed lower glucose consumption (Figure 3F). Moreover, glycerol production increased with differentiation time, and *PROS1* overexpression could decrease glycerol production (Figure 3G). Our results indicate that a low expression of *PROS1* may affect glucose metabolism and glycerol metabolism by activating a PPARγ signal and by facilitating preadipocyte differentiation.

### 3.4. Association between SNPs in PROS1 5′ Flanking Region and Chicken Growth Traits

The 5′ flanking region (NC_006088.5: 91130895-91132555) of *PROS1*, for which the length is 1661 bp, was amplificated to screen for polymorphisms. A total of eight genetic variations were obtained in the *PROS1* 5′ flanking region from a F2 population of Sanhuang chickens (*n* = 288). Subsequently, the eight genetic variations were identified in the *Ensembl* Database (Table 1). The eight genetic variations were all single nucleotide mutations (Figure S2). All genotypes of the alleles were classified according to their mutations. These SNPs’ allele information is listed in Table 1. A chi-square test was used to analysis whether allelic frequency meets the Hardy–Weinberg equilibrium. The allelic frequency of two SNPs (rs736154944 and rs739467563) deviated (*p*-value < 0.05). We speculated that it was caused by breeding selection.

The carcass traits were measured from the F2 population used in the genotype association analysis, including body weight (BW), dressed weight (DW), full-eviscerated weight (FEW), half-eviscerated weight (HEW), shin length (SL), shin circumference (SC), leg muscle weight (LMW), breast muscle weight (BMW), subcutaneous fat thickness (SFT), intermuscular fat width (IFT), abdominal fat weight (AFW), and abdominal fat weight rate (AFWR). To better understand the correlation among carcass traits, Pearson’s correlation coefficient was calculated. As shown in Table S2, the correlation between BW, DW, FEW, HEW, SL, SC, LMW, and BMW were all significant (*p* < 0.01), even though the correlation coefficients for SL and SC were lower. The correlation between the traits associated with fat (including SFT, IFW, AFW, and AFWR) were also all significant (*p* < 0.01). It is worth noting that AFW negatively correlated with BW, DW, FEW, HEW, SL, SC, LMW, and BMW, especially with SL and SC, indicating a potential auxiliary role of SL and SC on AFWR judgment.

Subsequently, an association analysis between the genotypes and carcass traits was performed following the general linear model (GLM) analysis. The SNPs that were significantly associated (*p* < 0.05) with a trait or that differed between phenotypes are listed in Table 2. As shown in the table, seven SNPs were associated with carcass traits, especially with fat-related traits. Considering a reduction in AFWR in the chicken breeding industry, rs740370863, rs736154944, rs739467563, rs313239277, and rs317295547 were favorable mutations, and rs731399734 and rs317681540 were undesirable mutations. Briefly, the CC genotype in rs740370863; the TT genotype in rs731399734, rs736154944, rs739467563, and rs317681540; and the AA genotype in rs313239277 and rs317295547 showed lower AFWR, indicating a potential benefit of reducing AFWR in chicken breeding.

## 4. Discussion

Abdominal fat deposition is involved with multiple biological processes, including preadipocyte proliferation and differentiation [16]. In this study, *PROS1* was considered the mechanism behind abdominal fat deposition and its specific roles in modulating preadipocyte proliferation and differentiation were characterized. Moreover, the SNPs in the 5′ flanking region showed a significant association with carcass traits.

*PROS1* has been widely identified as playing an essential role in the resolution of inflammation together with TAM receptor tyrosine kinases. In fact, the most obvious feature of *PROS1* is its TAM receptor-independent anticoagulation [17,18]. In our previous study, PRSO1 was upregulated in abdominal fat tissues from the individuals with low-abdominal fat rates [15], implying its potential role in chicken lipid metabolism. However, no studies on chicken *PROS1* have been reported. Here, we report that adipose tissue may be a major expression site for *PROS1* in chicken. Resnyk et al. found that the adipogenesis and lipogenesis genes were highly expressed in high-abdominal fat chicken, but the genes involved in the blood-coagulation pathway were highly expressed in low-abdominal fat chicken [19]. In addition, the affection of blood-coagulation protein levels caused by DNA polymorphisms in the blood-coagulation genes of lean chicken may be the main reason for high-expressions of blood-coagulation genes in low-fat chickens [16,20]. As a member involved in the blood-coagulation pathway, *PROS1* expression in low-abdominal fat individuals was higher than that in high-abdominal fat individuals, which was in agreement with the viewpoint of Resnyk.

Similarly, *PROS1* was reduced during preadipocyte differentiation, which again suggests a potential regulatory role of *PROS1* in adipogenesis., Adipocytes are a major source of free fatty acids in mammals, but the liver is the main site for de novo lipogenesis in avian animals [21,22]. However, adipocytes sourced from abdominal fat may contribute more to fatty synthesis in avian than previous perceptions [15,23,24]. T cell proliferation and function could be modulated by *PROS1*-dependent macrophages [25]. Here, we demonstrated that chicken *PROS1* promotes cell cycle progression to promote preadipocyte proliferation in vitro. A high expression of *PROS1* may play an essential role in weight maintenance after body weight loss [14], suggesting a negative effect of chicken *PROS1* in abdominal fat deposition. Subsequently, we confirmed the inhibition of *PROS1* on lipid droplet formation during the differentiation process from preadipocytes to adipocytes. Furthermore, glucose utilization and glycerol production were suppressed in preadipocytes under *PROS1* ectopic expression, indicating the substantial inhibition of *PROS1* on chicken preadipocyte differentiation. The chicken transcription factors PPARγ and CEBPα are both most important regulatory factors in abdominal fat preadipocyte development [26,27]. PPARγ was upregulated in high-fat chicken compared with low-fat chicken [28], and it was increased during the preadipocyte differentiation process [8]. The downregulation of both PPARγ mRNA and protein levels caused by *PROS1* suggests that the inhibition of *PROS1* on chicken preadipocyte differentiation may be mediated by the PPARγ signal. In brief, *PROS1* could inhibit chicken adipogenesis by downregulating the PPARγ signal.

It was reported that DNA polymorphisms of blood-coagulation genes in leaner chicken could affect their protein levels [16,20]. In this study, we explored the polymorphisms of *PROS1* in the 5′ flanking region. Although the SNPs in the gene 5′ flanking region could not cause amino acid sequence changes, synonymous mutations may regulate gene expression by affecting gene transcription by changing the DNA sequence of transcriptional regulatory sites [29,30]. Interestingly, the association between the seven SNPs located in the *PROS1* 5′ flanking region and abdominal fat traits showed that they may have more important effects on regulating abdominal fat tissue development.

## 5. Conclusions

In conclusion, the chicken *PROS1* gene facilitates preadipocyte proliferation and suppresses preadipocyte differentiation via the PPARγ signal, and the SNPs in the *PROS1* 5′ flanking region are significantly associated with the chicken abdominal fat weight rate, thereby suggesting a potential role of *PROS1* in chicken abdominal fat deposition.

## Figures and Tables

**Table 1:** The allele information of SNPs in the *PROS1* 5′ flanking region.

SNPs	Position in Genome	Allele	Genotypes of Alleles			Allelic Frequency	*p*-Value
rs740370863	NC_006088.5: 91131134	T→C	TT (*n* = 146)	TC (*n* = 127)	CC (*n* = 15)	T = 0.727 C = 0.273	0.057
rs731399734	NC_006088.5: 91131136	T→C	TT (*n* = 50)	TC (*n* = 144)	CC (*n* = 194)	T = 0.424 C = 0.576	0.685
rs736154944	NC_006088.5: 91131140	G→T	GG (*n* = 145)	GT (*n* = 128)	TT (*n* = 15)	G = 0.726 T = 0.274	0.048
rs739467563	NC_006088.5: 91131187	A→T	AA (*n* = 148)	AT (*n* = 127)	TT (*n* = 13)	A = 0.734 T = 0.266	0.027
rs317681540	NC_006088.5: 91131298	T→C	TT (*n* = 13)	TC (*n* = 111)	CC (*n* = 164)	T = 0.238 C = 0.762	0.284
rs315810115	NC_006088.5: 91131319	G→A	GG (*n* = 13)	GA (*n* = 111)	AA (*n* = 164)	T = 0.238 C = 0.762	0.284
rs313239277	NC_006088.5: 91131349	G→A	GG (*n* = 10)	GA (*n* = 106)	AA (*n* = 172)	G = 0.219 A = 0.781	0.192
rs317295547	NC_006088.5: 91131410	G→A	GG (*n* = 144)	GA (*n* = 126)	AA (*n* = 18)	G = 0.719 A = 0.281	0.163

**Table 2:** Associations between *PROS1* 5′ flanking region SNPs and carcass traits.

Traits	Genotypes (Number)			*p*-Value
rs740370863	CC (*n* = 15)	TC (*n* = 127)	TT (*n* = 146)	
IFW	24.33 ± 10.66 ^ab^	24.40 ± 10.10 ^a^	28.13 ± 9.73 ^b^	0.007
AFW	24.83 ± 12.59 ^a^	29.79 ± 16.73 ^a^	39.31 ± 15.93 ^b^	0.000
AFWR	1.87 ± 0.91 ^a^	2.30 ± 1.22 ^a^	3.07 ± 1.215 ^b^	0.000
rs731399734	CC (*n* = 94)	TC (*n* = 144)	TT (*n* = 50)	
AFW	37.31 ± 15.30 ^a^	33.67 ± 18.14 ^ab^	30.80 ± 15.33 ^b^	0.069
AFWR	2.93 ± 1.11 ^a^	2.61 ± 1.39 ^ab^	2.34 ± 1.09 ^b^	0.022
rs736154944	GG (*n* = 145)	GT (*n* = 128)	TT (*n* = 15)	
IFW	28.14 ± 9.66 ^a^	24.41 ± 10.18 ^b^	24.33 ± 10.66 ^ab^	0.007
AFW	39.48 ± 15.95 ^a^	29.67 ± 16.61 ^b^	24.83 ± 12.59 ^b^	0.000
AFWR	3.08 ± 1.22 ^a^	2.29 ± 1.21 ^b^	1.87 ± 0.91 ^b^	0.000
rs739467563	AA (*n* = 148)	AT (*n* = 127)	TT (*n* = 13)	
FEW	1246.18 ± 169.95 ^a^	1265.13 ± 151.95 ^ab^	1346.32 ± 191.57 ^b^	0.092
HEW	1493.74 ± 196.33 ^a^	1509.96 ± 177.35 ^ab^	1605.09 ± 227.01 ^b^	0.123
SL	75.06 ± 5.14 ^a^	76.36 ± 5.54 ^b^	76.92 ± 4.79 ^ab^	0.093
IFW	27.81 ± 9.74 ^a^	24.53 ± 10.24 ^b^	26.02 ± 10.37 ^ab^	0.026
AFW	38.81 ± 15.74 ^a^	30.02 ± 17.18 ^b^	26.03 ± 12.98 ^b^	0.000
AFWR	3.04 ± 1.21 ^a^	2.31 ± 1.23 ^b^	1.94 ± 0.97 ^b^	0.000
rs317681540	CC (*n* = 164)	TC (*n* = 111)	TT (*n* = 13)	
FEW	1246.18 ± 169.95 ^a^	1265.13 ± 151.95 ^ab^	1346.32 ± 191.57 ^b^	0.092
HEW	1493.74 ± 196.33 ^a^	1509.96 ± 177.35 ^ab^	1605.09 ± 227.01 ^b^	0.123
SL	75.06 ± 5.14 ^a^	76.36 ± 5.54 ^b^	76.92 ± 4.79 ^ab^	0.093
IFW	27.81 ± 9.74 ^a^	24.53 ± 10.24 ^b^	26.02 ± 10.37 ^ab^	0.026
AFW	38.81 ± 15.74 ^a^	30.02 ± 17.18 ^b^	26.03 ± 12.98 ^b^	0.000
AFWR	3.04 ± 1.21 ^a^	2.31 ± 1.23 ^b^	1.94 ± 0.97 ^b^	0.000
rs313239277	AA (*n* = 176)	GA (*n* = 106)	GG (*n* = 10)	
AFWR	2.53 ± 1.22 ^a^	2.87 ± 1.32 ^b^	2.81 ± 1.31 ^b^	0.094
rs317295547	AA (*n* = 18)	GA (*n* = 126)	GG (*n* = 144)	
IFW	25.80 ± 10.45 ^ab^	24.15 ± 10.03 ^a^	28.21 ± 9.77 ^b^	0.004
AFW	24.82 ± 11.84 ^a^	29.82 ± 16.76 ^a^	39.52 ± 15.93 ^b^	0.000
AFWR	1.90 ± 0.89 ^a^	2.30 ± 1.22 ^a^	3.08 ± 1.22 ^b^	0.000

Note: a significant difference is shown in superscript letters, with different letters representing a significant difference.

## Data Availability

The data presented in this study are available from the corresponding author upon request.

## Supplementary Materials

The following supporting information can be downloaded at https://www.mdpi.com/article/10.3390/ani12162046/s1. Figure S1: Original western blot figures for Figure 3D,E; Figure S2: *PROS1* 5’ flank region DNA polymorphism. The characters on the left represent the genotypes of each SNP. Every SNP site was ringed up by red rectangle; Table S1: Primers’ sequence information; Table S2: Correlation coefficients between carcass traits.
