# Supplementary material for: Chicken Protein S Gene Regulates Adipogenesis and Affects Abdominal Fat Deposition
Source: Animals (Basel). 2022 Aug 11;12(16):2046. doi: 10.3390/ani12162046 (PMC9404415; doi:10.3390/ani12162046)
Supplement: Supplementary file 1 [file animals-12-02046-s001.zip › animals-1708796-supplementary.pdf]

Supplementary Materials

Supplementary Figures:

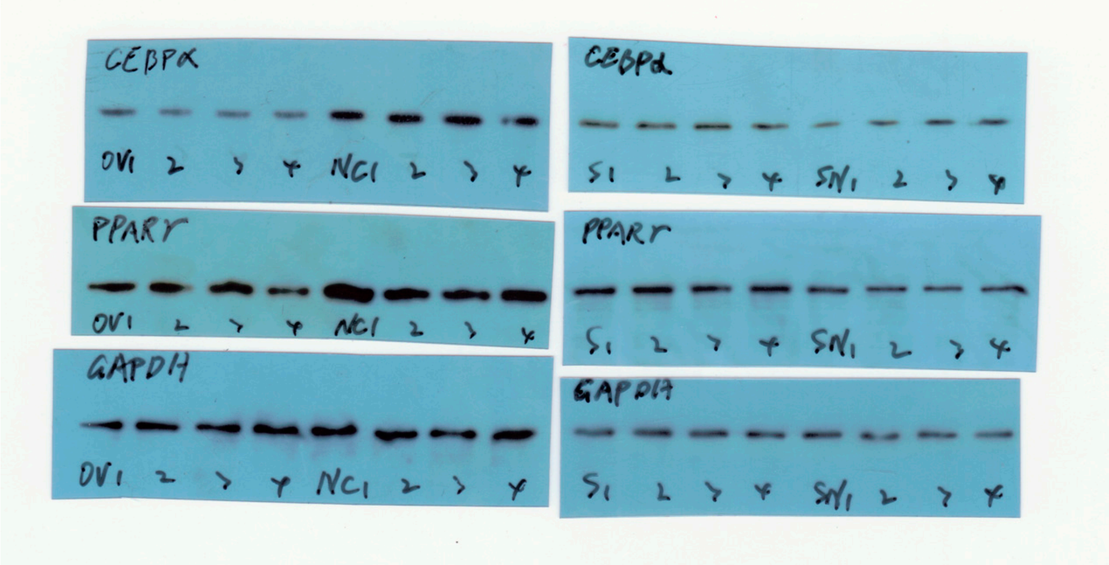

Figure S1. Original western blot figures for figure 3(D, E).

Note: OV 1-4 lanes represent 4 samples in PROS1 overexpression group, NC 1-4 lanes represent 4 samples in pcDNA3.1 group; S 1-4 lanes represent 4 samples in si-PROS1 group, SN 1-4 lanes represent 4 samples in si-NC group. The bands from top tp bottom were CEBP-alpha, PPAR-gama and GAPDH, respectively.

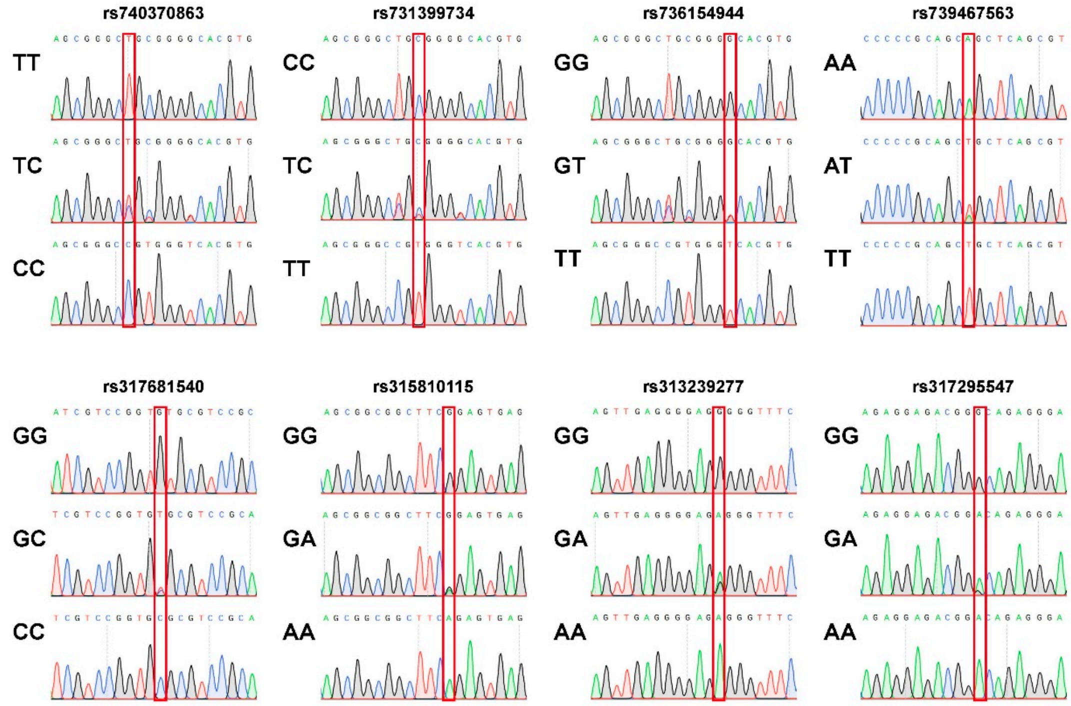

Figure S2. ***PROS1*** **5′ flank region DNA polymorphism.** The characters on the left represent the genotypes of each SNP. Every SNP site was ringed up by red rectangle.

Supplementary Tables:

Table S1. Primers' sequence information.

Primers' name	Sequence (5'-3')	Annealing temperature (°C)
q-*PROS1*-F	GCAGCGATGAGGCCG	59
q-*PROS1*-R	TCAGAGGCATACTGGTGGGA	
q-Cyclin D1-F	CAGAAGTGCGAAGAGGAAGT	58
q-Cyclin D1-R	CTGATGGAGTTGTCGGTGTA	
q-Cyclin D2-F	AACTTGCTCTACGACGACC	58
q-Cyclin D2-R	TTCACAGACCTCCAACATC	
q-PCNA-F	GTGCTGGGACCTGGGTT	58
q-PCNA-R	CGTATCCGCATTGTCTTCT	
q-Cyclin B2-F	CAGTAAAGGCTACGAAAG	58
q-Cyclin B2-R	ACATCCATAGGGACAGG	
q-CDKN1B-F	GCTGTGCTGGGCTGAA	58
q-CDKN1B-R	GGACGAAAGGATGTGGG	
q-p21-F	GAAGAGTTGTCCACGATAAGC	58
q-p21-R	TTCCAGTCCTCCTCAGTCC	
q-PPARγ-F	TCCTTCCCGCTGACCAAA	60
q-PPARγ-R	TCCTGCACTGCCTCCACA	
q-ADIPOR1-F	GACAAGAACAGCAACGAGTACCGC	60
q-ADIPOR1-R	CCTGAAGATGCCCCGCAGAGT	
q-CEBPα-F	GACAAGAACAGCAACGAGTACCGC	56
q-CEBPα-R	CCTGAAGATGCCCCGCAGAGT	
q-CEBPβ-F	GCGGACTGTTTGGCTGCTCT	60
q-CEBPβ-R	CGGGTGAGGCTGATGTAGGTGT	
q-LPL-F	CCAAGGTAGACCAGCCATTC	60
q-LPL-R	TGCTCCAGGCACTTCACA	
q-GAPDH-F	CAACTTTGGCATTGTGGAGG	56
q-GAPDH-R	CGCTGGGATGATGTTCTGG	
PROS1-5' flank-F	AGTTACCACAGAAACTAAGAAAACC	58
PROS1-5' flank-R	CACCTCTTCGCCGCTGAT	

Table S2. Correlation coefficients between carcass traits.

	BW	DW	FEW	HEW	LMW	BMW	SL	SC	SFT	IFW	AFW	AFWR
BW	1											
DW	0.956*	1										
FEW	0.952*	0.957*	1									
HEW	0.961*	0.965*	0.991*	1								
LMW	0.903*	0.912*	0.933*	0.933*	1							
BMW	0.768*	0.776*	0.818*	0.796*	0.737*	1						
SL	0.548*	0.549*	0.556*	0.571*	0.636*	0.346*	1					
SC	0.496*	0.476*	0.484*	0.506*	0.527*	0.236*	0.478*	1				
SFT	-0.009	0.004	0.03	0.029	-0.036	0.081	-0.153*	-0.091	1			
IFW	0.225*	0.228*	0.228*	0.236*	0.167*	0.181*	-0.03	-0.063	0.109	1		
AFW	0.106	0.097	0.097	0.115	-0.019	0.149*	-0.297*	-0.237*	0.234*	0.433*	1	
AFWR	-0.132*	-0.142*	-0.153*	-0.131*	-0.242*	-0.074	-0.407*	-0.345*	0.216*	0.372*	0.960*	1

Note: BW: body weight; DW: dressed weight; FEW: full-eviscerated weight; HEW: half-eviscerated weight; SL: shin length; SC: shin circumference; LW: leg muscle weight; BMW: breast muscle weight; SFT: Subcutaneous fat thickness; IFW: intermuscular fat width; AFW: abdominal fat weight; AFWR:

abdominal fat weight rate. Correlation coefficients were calculated by Pearson's analysis in IBM SPSS v25.0. Two-tailed T test was used in significance analysis, *: $p < 0.05$.
